# The epigenetic modifier CHD5 functions as a novel tumor suppressor for renal cell carcinoma and is predominantly inactivated by promoter CpG methylation

**DOI:** 10.18632/oncotarget.7822

**Published:** 2016-03-01

**Authors:** Zhenfang Du, Lili Li, Xin Huang, Jie Jin, Suming Huang, Qian Zhang, Qian Tao

**Affiliations:** ^1^ Cancer Epigenetics Laboratory, Department of Clinical Oncology, State Key Laboratory of Oncology in South China, Sir YK Pao Center for Cancer and Li Ka Shing Institute of Health Sciences, The Chinese University of Hong Kong and CUHK-Shenzhen Research Institute, Shatin, Hong Kong; ^2^ Department of Urology, Peking University First Hospital and Institute of Urology, National Research Center for Genitourinary Oncology, Beijing, China; ^3^ Departments of Biochemistry and Molecular Biology, University of Florida College of Medicine, Gainesville, Florida, USA

**Keywords:** CHD5, tumor suppressor, methylation, renal cell carcinoma, oncogene

## Abstract

Renal cell carcinoma (RCC) is the most common urological cancer with steadily increasing incidence. A series of tumor suppressor genes (TSGs) have been identified methylated in RCC as potential epigenetic biomarkers. We identified a 1p36.3 TSG candidate *CHD5* as a methylated target in RCC through epigenome study. As the role of *CHD5* in RCC pathogenesis remains elusive, we further studied its expression and molecular functions in RCC cells. We found that *CHD5* was broadly expressed in most normal genitourinary tissues including kidney, but frequently silenced or downregulated by promoter CpG methylation in 78% of RCC cell lines and 44% (24/55) of primary tumors. In addition, *CHD5* mutations appear to be rare in RCC tumors through genome database mining. In methylated/silenced RCC cell lines, *CHD5* expression could be restored with azacytidine demethylation treatment. Ectopic expression of *CHD5* in RCC cells significantly inhibited their clonogenicity, migration and invasion. Moreover, we found that CHD5, as a chromatin remodeling factor, suppressed the expression of multiple targets including oncogenes (*MYC*, *MDM2*, *STAT3*, *CCND1*, *YAP1*), epigenetic master genes (*Bmi-1*, *EZH2*, *JMJD2C*), as well as epithelial-mesenchymal transition and stem cell markers (*SNAI1*, *FN1, OCT4*). Further chromatin immunoprecipitation (ChIP) assays confirmed the binding of CHD5 to target gene promoters. Thus, we demonstrate that *CHD5* functions as a novel TSG for RCC, but is predominantly inactivated by promoter methylation in primary tumors.

## INTRODUCTION

Renal cell carcinoma (RCC) is a common cancer which accounts for ∼90% of kidney cancer cases in adults, with over 200,000 cases worldwide per year [1] and its incidence steadily rising in most areas of the world [2, 3]. Despite of recent advances in RCC diagnosis and therapy, many patients are still present with metastasis at initial diagnosis and poor treatment response rates [4]. Therefore, elucidation of the molecular mechanisms underlying RCC development and progression is urgently needed [5].

Cancer is caused by cumulative genetic and epigenetic alterations [6]. Epigenetic silencing of tumor suppressor genes (TSGs) through promoter CpG methylation confers selective advantages of clonal expansion, leading to tumor initiation and progression [7]. Promoter methylation of TSGs can also be used as epigenetic biomarkers for tumor diagnosis [8]. A series of genes have been identified to be methylated in RCC with some of them linked to tumor prognosis [9]. For example, *VHL* is one of TSGs early identified to be epigenetically inactivated by promoter CpG methylation in RCC [10]. *RASSF1A*, which maps to the 3p21 region of frequent allele loss, was methylated in 30∼50% of sporadic clear cell (cc) RCC and papillary RCC [11, 12]. *SLC16A3* promoter methylation is a predictive marker for the prognosis and clinical outcome of ccRCC [13]. Our group has also identified several novel TSGs silenced by promoter methylation in RCC, including *DLC1*, *DLEC1* and *IRF8*. The methylation of these TSGs is associated with patient poor prognosis, thus as potential biomarkers for RCC [14–16].

Chromodomain helicase DNA binding (CHD) genes encode a class of ATPase-dependent DNA-binding proteins interacting with histones to modulate chromatin structure and transcription [17]. *CHD5*, located at 1p36.3, is the fifth member of a nine-member family of CHD chromatin remodeling proteins (CHD1-CHD9) [18]. CHD5 consists of two tandem plant homeodomains (PHDs), dual chromodomains, SNF2 family N-terminal domain (SNF2N), ATP-dependent helicase conserved C-terminal domain (HELIC) followed by other domains with unknown functions [19–21]. *CHD5* has been reported frequently methylated in multiple human cancers, including glioma, breast, lung, gastric, colon, ovarian and prostate cancers [18, 22]. Reduced *CHD5* expression is associated with unfavorable clinical features and outcome of cancer patients [23–26]. In mice, Chd5 functions as a dose-dependent TSG through regulating cell proliferation, apoptosis and senescence, due to upregulation of p19^Arf^ and TP53 [27]. CHD5 significantly inhibits clonogenic growth and tumor xenograft growth, thus as a functional tumor suppressor in multiple common cancers, including breast, colon, lung, ovary and prostate cancers [18], although no report about the expression and function of *CHD5* has been reported in RCC yet.

We have identified *CHD5* as a methylated target in RCC cell lines. Here, we further studied the epigenetic alteration of *CHD5* in RCC cells, and characterized its tumor suppressive functions and the underlying molecular mechanisms during RCC pathogenesis.

## RESULTS

### Identification of *CHD5* as a methylated TSG candidate for urological cancers

**Figure 1:**
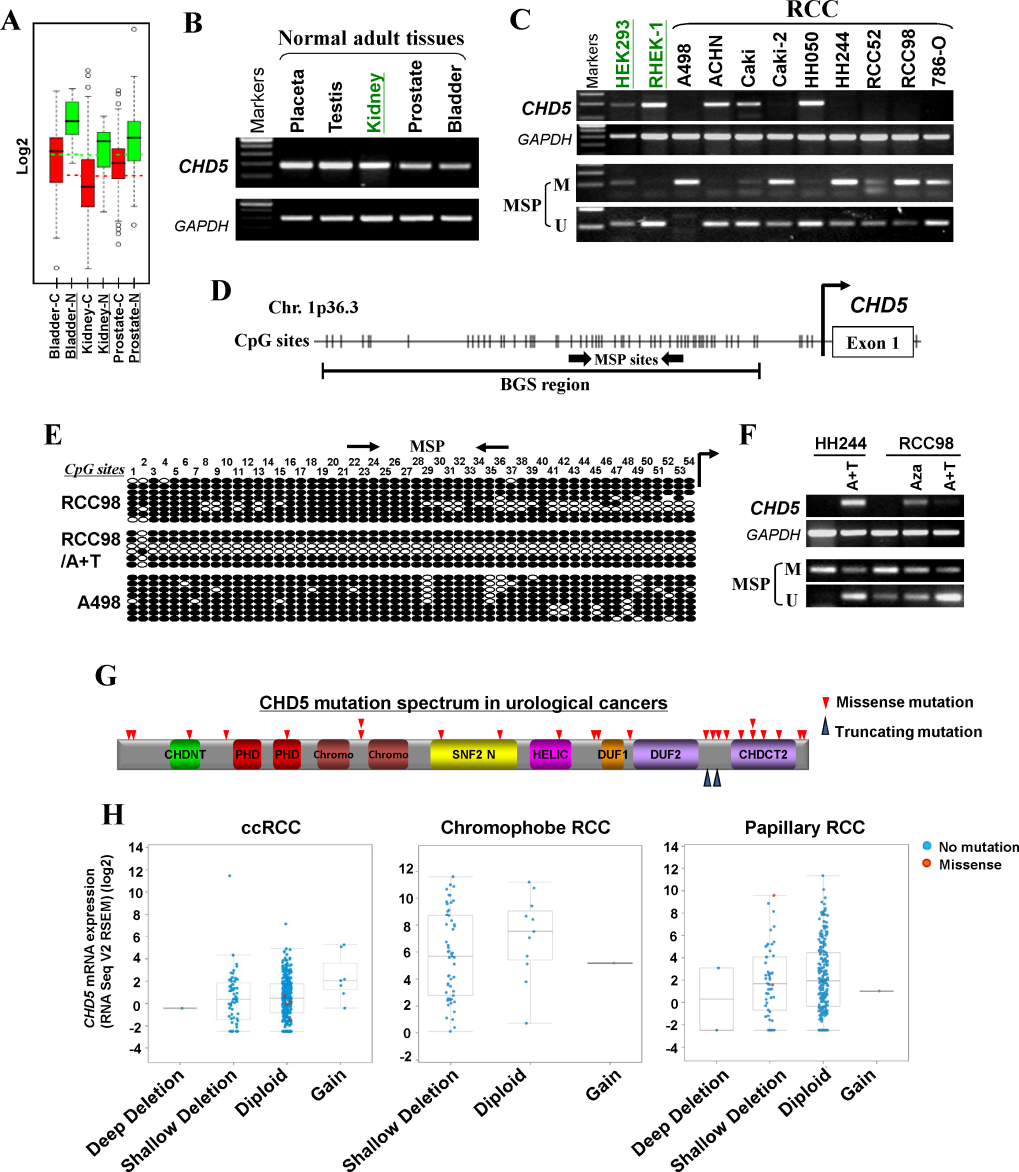
*CHD5* is predominantly inactivated by promoter CpG methylation in urological cancers (**A**) Expression profiles of *CHD5* in urological tumors and normal tissues. C, cancer; N, normal (GENT datasets, http://medical-genome.kribb.re.kr/GENT/search/search.php). (**B**) *CHD5* expression in a panel of human normal adult tissues detected by RT-PCR, with *GAPDH* as an internal control. (**C**) *CHD5* was downregulated or silenced by promoter methylation in RCC cell lines as determined by RT-PCR and MSP, but expressed and unmethylated in HEK293 and RHEK-1 cell line. M, methylated; U, unmethylated. (**D**) Schematic structure of the *CHD5* promoter region. Exon 1, CpG sites (short vertical lines), MSP sites and BGS region analyzed are shown. (**E**) BGS analysis of the *CHD5* promoter in representative RCC cells. Each row of circles represented an individual promoter allele. Filled circle, methylated CpG site; open circle, unmethylated CpG site. (**F**) Pharmacologic demethylation with Aza alone or combined with TSA (A + T) restored *CHD5* expression in methylated/silenced tumor cell lines. (**G**) Schematic representation of *CHD5* somatic mutations identified in urological cancers. CHDNT, CHD N-terminal domain; PHD, plant homeodomain; Chromo, CHRromatin Organisation MOdifier domain; SNF2 N, SNF2 family N-terminal domain; HELIC, Helicase conserved C-terminal domain; DUF, Domain of Unknown Function; CHDCT2, CHD C-terminal domain. (**H**) *CHD5* putative copy number alterations from GISTIC (Genomic Identification of Significant Targets in Cancer): loss of an allele of CHD5 correlated with decreased mRNA expression in ccRCC (left), chromophobe RCC (middle) and papillary RCC (right).

We have previously identified *CHD5* as a methylated target through epigenome study [28]. Meanwhile, through analyzing microarray data from GENT dataset [29], we found that *CHD5* was underexpressed in 366 kidney cancer tissues, 87 bladder cancer tissues and 244 prostate cancer tissues, compared with the corresponding normal tissues (Figure 1A). Data from Oncomine database [30] also indicated that *CHD5* mRNA expression was frequently decreased in kidney [31–33], bladder [34] and prostate cancers [35] compare with their adjacent control tissues (Supplementary Table 1). Our semi-quantitative RT-PCR data showed that *CHD5* was silenced or downregulated in 7/9 RCC, 2/3 prostate and 1/3 bladder tumor cell lines, but readily detected in most human normal adult tissues including kidney and prostate, as well as immortalized normal cell lines (HEK293 and RHEK-1) (Figure 1B and 1C, Supplementary Figure 1A). Then we analyzed the promoter region of *CHD5*, and found that there was a typical CpG island spanning the transcription start site (Figure 1D). These results indicated that *CHD5* is a downregulated candidate TSG for urological cancers and possibly subjected to methylation-mediated silencing.

### Silencing of *CHD5* due to its promoter CpG methylation in urological cancers

We next studied whether promoter CpG methylation was involved in silencing *CHD5* in urological cancers. Methylation-specific PCR (MSP) analysis showed that *CHD5* was frequently methylated in RCC, prostate and bladder tumor cell lines, which was negatively correlated with the corresponding expression levels (Figure 1C, Supplementary Figure 1A). To examine the methylation status of *CHD5* promoter in more detail, bisulfite genomic sequencing (BGS) analysis was performed for a 539-bp region with 54 CpG sites spanning the *CHD5* core promoter and exon 1. High density of methylated CpG sites were detected in two representative RCC cell lines (RCC98 and A498) and one prostate tumor cell line (PC3), which confirmed the MSP data (Figure 1E, Supplementary Figure 1B).

To determine whether methylation directly contributes to *CHD5* silencing, we treated two RCC cell lines (HH244 and RCC98) with DNA methyltransferase inhibitor Aza, alone or in combination with histone deacetylase inhibitor TSA. After pharmological treatment, *CHD5* expression was restored in HH244 and RCC98 cells, accompanied by significant increase of unmethylated alleles and decrease of methylated alleles (Figure 1F). Demethylation of *CHD5* promoter in RCC98 cells was confirmed by BGS analysis (Figure 1E).

Moreover, the data retrieved from cBio database [36, 37] revealed the presence of copy number loss of *CHD5* in ccRCC [31], papillary RCC (TCGA) and prostate cancer [35, 38, 39] (Supplementary Figure 2). Meanwhile, the data also indicated that *CHD5* was rarely mutated in urological cancers [40–42] (Figure 1G and Supplementary Table 2). Loss or downregulation of *CHD5* expression correlated with copy number loss using data from the TCGA RCC cohorts (Figure 1H). Together, these findings suggested *CHD5* as a candidate TSG that was epigenetically silenced in urological cancers.

### Frequent methylation of *CHD5* in primary RCC tumors

**Figure 2:**
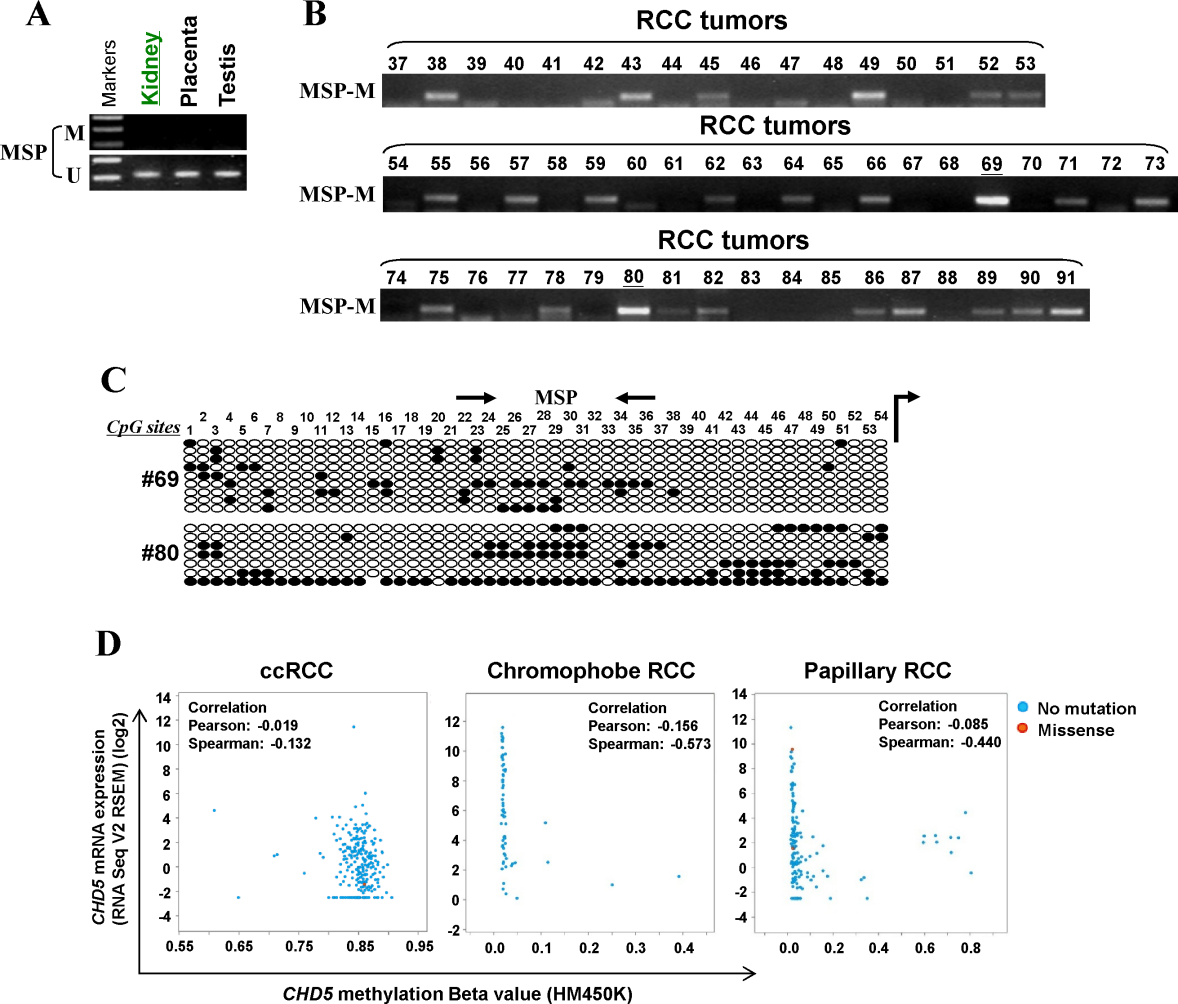
*CHD5* is methylated in primary RCC tissues (**A**) MSP analysis of *CHD5* methylation in normal urological tissues. (**B**) Representative MSP analysis of *CHD5* methylation in RCC tissues. All these samples have been shown to have sufficient bisulfite converted DNA by unmethylation detection for TSGs. (**C**) BGS analysis confirmed *CHD5* methylation in two representative primary RCC tissues. (**D**) *CHD5* methylation was observed in all of ccRCC (320/320), 3.0% (2/66) chromophobe RCC and 4.1% (12/292) in papillary RCC primary tumors from TCGA RCC cohorts.

Through MSP analysis, we found that normal urological tissues exhibited unmethylated *CHD5* promoter (Figure 2A). Meanwhile, we examined *CHD5* methylation in our RCC samples. MSP analysis showed that *CHD5* was methylated in 44% (24/55) of RCC samples (Figure 2B). High-resolution BGS analysis of two representative cases further confirmed the methylation (Figure 2C). The frequency of *CHD5* methylation in our patients group was comparable to the data obtained from The Cancer Genome Altas (TCGA) RCC cohorts. The overall frequency of *CHD5* methylation in TCGA RCC cohorts was 49% (334/688), with highest in ccRCC (100%, 320/320) [31], followed by papillary RCC (4.1%,12/292) and chromophobe RCC (3%, 2/66) [43] (Figure 2D).

### CHD5 suppresses RCC cell clonogenicity and induces apoptosis

**Figure 3:**
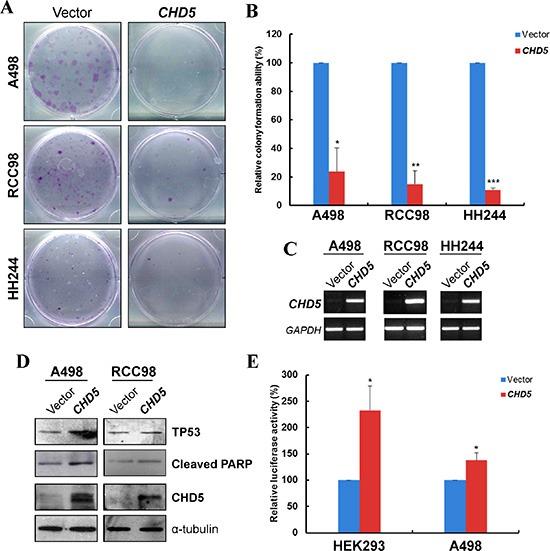
CHD5 suppresses RCC cell growth and induced TP53-related apoptosis (**A**) Representative monolayer culture colony formation assay of A498, RCC98 and HH244 cells. (**B**) Quantitative analysis of colony numbers. Data are presented as mean ± SD of three independent experiments. *P < 0.05, **P < 0.01 ***P < 0.001. (**C**) Restored expression of *CHD5* in transfected cell lines as confirmed by RT-PCR. (**D**) Western blot showed upregulation of cleaved PARP and TP53 in *CHD5*-expressing RCC cells. α-tubulin was used as a loading control. (**E**) *CHD5* expression can increase the transcription activity of TP53-binding site promoter construct as determined by luciferase reporter assay in HEK293 and A498 cell lines. *P < 0.05.

Next, we assessed the impact of *CHD5* on clonogenicity with monolayer cell colony formation assay in three RCC cell lines (A498, RCC98 and HH244). We transfected A498, RCC98 and HH244 cell line with empty vector or pcDNA3.1/*CHD5*, and the restored expression of *CHD5* was validated by semi-quantitative RT-PCR. Results showed that ectopic expression of *CHD5* significantly decreased the numbers of RCC cell colonies, compared with controls (Figure 3A, 3B and 3C).

As the growth inhibition could be attributed to the induction of apoptosis, we then examined the effect of CHD5 on the apoptosis of RCC cells. We observed the cleavage of poly (ADP-ribose) polymerase (PARP), a classic apoptotic marker in cells with ectopic expression of CHD5 (Figure 3D). Additionally, CHD5 has been reported to regulate TP53-induced apoptosis [27]. Hence we assessed the expression and activation of TP53 in *CHD5*-expressing RCC cells. As shown in Figure 3D, *CHD5* could upregulate TP53 expression in RCC cells. Moreover, through dual luciferase reporter assay of TP53 binding-site promoter construct, *CHD5*-expressing HEK293 and A498 cells showed significantly elevated TP53 transcriptional activities when compared with controls (Figure 3E). Collectively, the data indicated that *CHD5* could suppress the clonogenicity of RCC cells, which might be linked to the enhancement of TP53-induced apoptosis.

### CHD5 inhibits the migration and invasion of RCC cells

**Figure 4:**
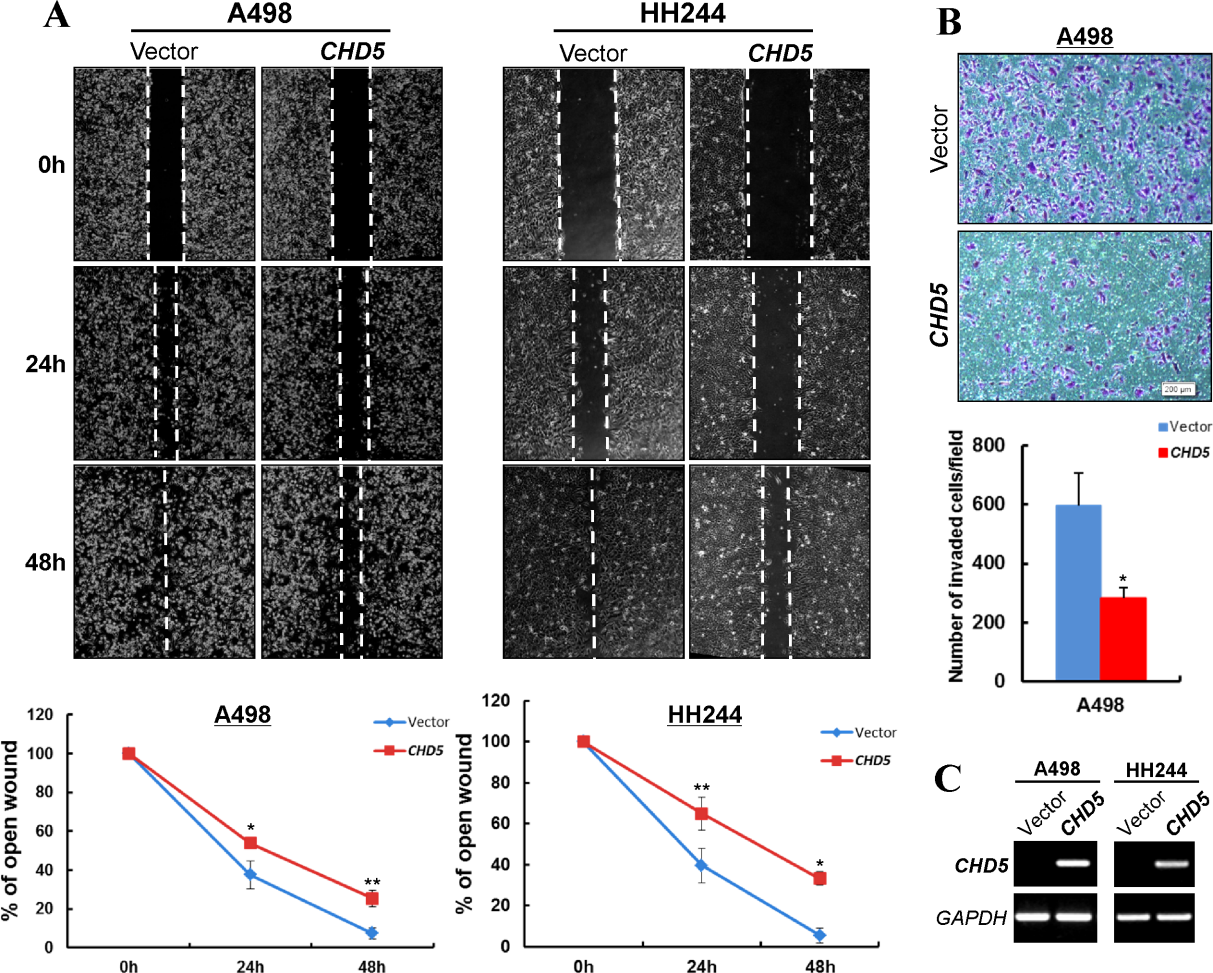
CHD5 inhibits the migration and invasion of RCC cells (**A**) Wound healing assay demonstrated a slower wound closure of *CHD5*-expressing A498 and HH244 cells compared with controls. *P < 0.05; **P < 0.01. (**B**) Transwell migration assay of *CHD5*-expressing tumor cells. Migrated cells at the lower surface of the transwell filter were stained (up panel) and counted (bottom panel). *P < 0.05. (**C**) Expression of *CHD5* in transfected cell lines was confirmed by RT-PCR.

We also assessed the effects of CHD5 on RCC cell migration and invasion. Scratch wound-healing assay showed that *CHD5*-expressing A498 and HH244 cells were less proficient in healing an artificial wound than the vector-transfected cells on a confluent monolayer (Figure 4A, 4C). Moreover, *CHD5*-expressing cells displayed significantly decreased invasiveness compared with controls in Matrigel invasion assay (Figure 4B, 4C).

### CHD5 represses the expression of multiple cancer genes through direct binding to their promoters

**Figure 5:**
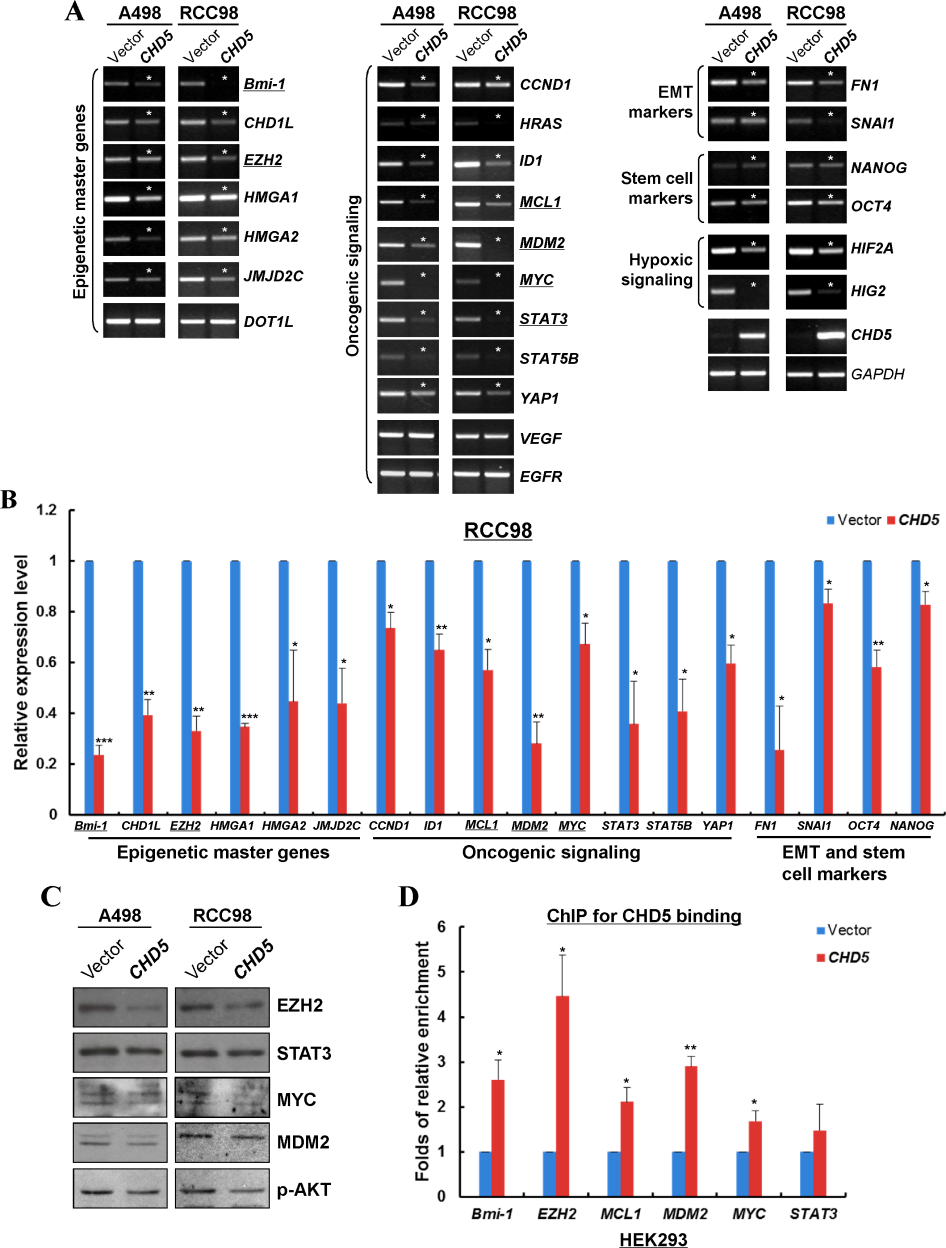
CHD5 represses the expression of multiple cancer genes through direct interaction with their promoters (**A**) Semi-quantitative RT-PCR showed that CHD5 repressed the mRNA expression of multiple oncogenic genes in A498 and RCC98 cells. Asterisk (*) indicates significantly downregulation effects. (**B**) qRT-PCR showed that CHD5 inhibited the expression levels of multiple cancer genes in RCC98 cells, including epigenetic master genes, hypoxia-inducible factors, oncogenic genes, EMT and stem cell markers. *P < 0.05; **P < 0.01; ***P < 0.001. (**C**) Western blot showed decreased levels of EZH2, STAT3, MYC, MDM2 and p-AKT by CHD5. Gel loading control is the same as in Figure 3D. (**D**) ChIP-qPCR showed enrichment of CHD5 binding to the promoter regions of *EZH2*, *MYC*, *MDM2*, *BMI1* and *MCL1* in HEK293 cell lines. *P < 0.05; **P < 0.01.

We further investigated the possible mechanism of CHD5 functioning as a tumor suppressor in RCC cells. We firstly examined the expression levels of multiple cancer genes in RCC cells with ectopic *CHD5* expression, including oncogenes, epigenetic master genes, epithelial-mesenchymal transition (EMT) and stem cell-related genes. Semi-quantitative and qRT-PCR showed that the expression levels of multiple oncogenes (*MYC*, *MDM2*, *STAT3*, *CCND1*, *YAP1* etc.), epigenetic master genes (*Bmi-1, EZH2, JMJD2C* etc.), epithelial-mesenchymal transition and stem cell markers (*FN1, SNAI1, OCT4* and *NANOG*) and hypoxia-inducible factors (*HIF2A* and *HIG2*) were significantly decreased in *CHD5*-transfected A498 and RCC98 cells (Figure 5A and 5B). Furthermore, we examined the protein expression of some cancer genes by Western blot. We found that the protein levels of EZH2, STAT3, MYC, MDM2 and p-AKT were decreased in *CHD5*-expressing RCC cells (Figure 5C). We further performed ChIP assay to detect the recruitments of CHD5 to *MYC*, *MDM2*, *EZH2*, *Bmi-1* and *MCL1* promoters. It was found that CHD5 indeed bound to these promoters with varied binding activities (Figure 5D).

## DISCUSSION

**Figure 6:**
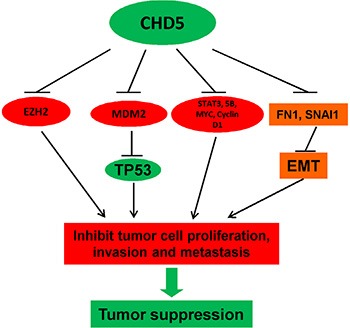
Proposed model of the tumor suppressive functions of CHD5 in RCC CHD5 promotes TP53-related apoptosis and suppresses multiple cancer gene expression (*EZH2*, *MYC* and *MDM2*, etc), leading further to tumor suppression.

In this study, we demonstrated that *CHD5* acted as a functional tumor suppressor and was frequently silenced by promoter CpG methylation in RCC. Recent findings suggest that genetic and epigenetic alterations are both involved in RCC development. Identification of epigenetic alterations in RCC could be helpful to unravel the mechanisms underlying RCC carcinogenesis and develop potential biomarkers for cancer screening and prognosis prediction [31]. *CHD5* is located at 1p36, together with other tumor suppressors including p73, CAMTA1, miR-34a, KIF1β, and CASZ1 [18]. *CHD5* is reported to be rarely mutated, but with frequent allelic loss in cancers. *CHD5* methylation has been identified in several cancer types including glioma, breast, colon, lung, ovary and prostate cancers [18, 22], suggesting the contribution of epigenetic regulation to its biallelic inactivation [18]. Our present data demonstrated that CHD5 could inhibit cell proliferation, migration and invasion, as well as induce apoptosis in RCC cells, supporting the involvement of epigenetic inactivation of *CHD5* in RCC tumorigenesis (Figure 6).

Nucleosome remodeling and deacetylation (NuRD) complex plays a critical role in the regulation of transcriptional events during normal physiology and cancer pathogenesis including RCC [17, 31]. CHD5, being a component of NuRD complex, has been identified to be capable of suppressing the transcription of cell-cycle regulator WEE1. CHD5 mutant lacking the ATPase activity due to mutation within HELIC domain maintained its interaction with other NuRD subunits, but the ability to repress *WEE1* transcription was decreased [44]. The dual PHDs of Chd5 specifically interacts with unmodified N-terminus of histone H3, which is ciritical for Chd5 to exert the regulatory role on gene expression and the potential tumor suppressive function [21, 45]. Therefore, both PHDs and HELIC are required for the tumor suppressive function of CHD5. Recently, Potts RC *et al.* demonstrated that CHD5 protein can regulate target genes through direct interaction in the form of a NuRD-like multi-protein complex [46]. Here, we demonstrated that CHD5 could repress the transcription of multiple oncogenes, including *MYC* and *EZH2*, through direct binding to their promoters.

In conclusion, *CHD5* is frequently silenced by promoter methylation in urological cancers including RCC, and functions as a TSG through direct repression of multiple oncogenes in RCC cells. Our present study emphasizes the contribution of epigenetic regulation to RCC carcinogenesis and silencing of *CHD5* could be a potential tumor biomarker for RCC diagnosis.

## MATERIALS AND METHODS

### Database analysis

Databases including GENT [29], Oncomine [30], cBio (MSKCC) [36, 37] and Catalogue of Somatic Mutations in Cancer (COSMIC) (Wellcome-Sanger) [47] database were screened for information specifying genomic alterations and mRNA expression in TCGA cohorts and other published papers. Functionality of *CHD5* mutation was evaluated by PolyPhen-2 software (Version 2.2.2) [48].

### Cell lines and tissue samples

RCC cell lines (A498, ACHN, Caki, Caki-2, HH050, HH244, RCC52, RCC98 and 786-O) were routinely maintained in RPMI1640 (Invitrogen, Grand Island, NY) with 10% fetal bovine serum. Immortalized, non-transformed normal epithelial cell line HEK293 and RHEK-1 (kindly gifted by Prof. John Rhim, US Naval Medical Research Center, Bethesda, MD) were used as controls. Cell lines were obtained from either the American Type Culture Collection or our collaborators. Human normal tissues RNA were purchased from Stratagene (Santa Clara, CA), Biochain (Newark, CA) or Chemicon (Billerica, MA). DNA samples of RCC cases were obtained from our collaborators as described previously [14].

### Semi-quantitative and quantitative RT-PCR

Total RNA was extracted using TRI reagent. Reverse transcription (RT) using random hexamer, and RT-PCR using Go-Taq (Promega, Madison, WI) were performed as previously described [49]. Primers used for RT-PCR were listed in Table 1 and Supplementary Table 3.

**Table 1:** Primers used for the detection of CHD5 expression and promoter methylation

Type	Primers	Sequence (5′–3′)	Length
RT-PCR	CHD5F	CCAGTGGGCACCGAGGAG	
	CHD5R	CTTCTTCCGCTTCCCTTTAC	192 bp
MSP	CHD5m1	GTTCGGGGTTTAGCGTTTTC	
	CHD5m2	GAAACTTAACGAACCCGAACG	108 bp
	CHD5u1	GGGTTTGGGGTTTAGTGTTTTT	
	CHD5u2	TCAAAACTTAACAAACCCAAACA	112 bp
BGS	CHD5BGS1	GGGTTTTAGTTGTATTTAGTTTG	
	CHD5BGS2	TAACAACAAAAAACAAATTAAAAAAC	539 bp

Quantitative real-time PCR (qRT-PCR) was performed as previously described [50]. SYBR Green master mix (Applied Biosystems, Grand Island, NY) was used. The expression levels of target genes in *CHD5*-transfected cells were normalized to those transfected with vector control. *GAPDH* was used as an internal control. The sequences of primers used in qRT-PCR were listed in Table 1 and Supplementary Table 3.

### Bisulfite treatment and promoter methylation analysis

Bisulfite modification of DNA, MSP and BGS analysis was conducted as previously described [49]. 38 cycles of PCR reaction were performed in MSP using primers amplifying methylated gene allele, with 40 cycles for reaction using primers amplifying unmethylated gene allele. For BGS, PCR products amplified using BGS primers were cloned into pCR4-TOPO vector (Invitrogen, Carlsbad, CA, USA), with 6–10 colonies randomly chosen and sequenced. Primers used for MSP and BGS were listed in Table 1.

### Demethylation treatment using 5-Aza-2′-deoxycytidine and trichostatin A

Treatment of RCC cell line using 5-Aza-2′-deoxycytidine (Aza) and trichostatin A (TSA) was carried out as previously described [51]. Briefly, cells were treated with 10 μM Aza (Sigma, Ronkonkoma, NY) for 72 hours and harvested for DNA and RNA extraction. Alternatively, after 72 hours of Aza treatment, cells were incubated with 100 ng/ml TSA for additional 24 hours.

### Construction of *CHD5* expression vector

The full-length Open Reading Frame (ORF) of *CHD5* was amplified and cloned to pcDNA3.1 (+) vector, with a Flag tag added to its N terminus. The sequence and orientation of *CHD5* ORF were confirmed by Sanger sequencing.

### Colony formation assays

A498, HH244 and RCC98 cells were seeded in a 12-well plate at 1∼2 × 10^5^/well. The cells were then transfected with *CHD5* plasmid or empty vector using Lipofectamine 2000 (Invitrogen). After 48 hours post-transfection, the transfectants were sub-cultured into 6-well plates for selection with 200 μg/ml G418 (Calbiochem, Darmstadt, Germany). After 1∼2 weeks of selection, surviving colonies (> 50 cells/colony) were fixed with methanol and stained with Gentian Violet and counted.

### Dual-luciferase reporter assay

TP53 transcriptional activity was determined by luciferase reporter assay. After 48 hours of transfection, luciferase activities were determined using a dual-luciferase reporter assay kit (Promega, Madison, WI, USA). Relative luciferase activities were determined and normalized using Renilla reniformis luciferase activity as an internal control.

### Wound healing assay and matrigel invasion assays

Wound healing assay to evaluate cell migration ability was performed as previously described [51]. Briefly, cells transfected with empty vector or *CHD5* construct were allowed to growth until confluent (> 95%). Cell scratches were then created in A498 and HH244 cell lines using 200 μl sterile tips and washed twice with culturing medium. After indicated time points of incubation, cells were imaged under a phase-contrast microscope. The experiments were performed in triplicate. *In-vitro* invasion assays were carried out in Corning BioCoat Matrigel chambers (Corning, NY) as described previously [51].

### Western blot

Western blot was performed as described previously [51]. Briefly, membranes were incubated with primary antibody at 4°C overnight, followed by incubation with secondary antibody at room temperature for 1 hour. Immunoreactive bands were detected using Western blot Luminol reagent (GE Healthcare, Waukesha, WI). Antibodies used were CHD5 (23320002, Novus), EZH2 (18-7395, Invitrogen), MDM2 (sc-813, Santa Cruz, CA), MYC (sc-764, Santa Curz, CA), cleaved poly (ADP-ribose) polymerase (9541, Cell Signal), phospho-AKT (4060, Cell Signal), α-tubulin (MS-581, Thermo Lab Vision, MI); p53 (M7001), anti-mouse IgG-HRP (P0161), anti-rabbit IgG-HRP (P0448) (Dako, Glostrup, Denmark).

### Chromatin immunoprecipitation

Chromatin immunoprecipitation (ChIP) was carried out as previously described using ChIP-IT Express Kit from Active Motif (53008; Carlsbad, CA) [50]. For each ChIP reaction, 20 μg of total chromatin was incubated with 20 μl of Protein G magnetic beads and 1 μg of FLAG-M2 antibody (F3165; Sigma) at 4°C overnight. Both input and precipitated DNA were purified with QIAamp DNA Mini Kit (Qiagen, Valencia, CA) for subsequent quantitative real-time PCR (ChIP-qPCR). The relative enrichment of precipitated DNA was normalized to normal mouse IgG. Primers used for ChIP assay were listed in Table 2 and the locations of primers were shown in Supplementary Figure 3

**Table 2:** Primers for ChIP-qPCR used in this study

Gene	Primers	Sequence (5′–3′)	Length (bp)
*Bmi-1*	BMI1ChIPF4	TCTCTGCAATTTGAGCCCTG	205
	BMI1ChIPR4	GAAAATGCAAACCGCACTCC	
*EZH2*	EZH2ChIPF1	AAATTAGTCGGGTGTGGTGG	152
	EZH2ChIPR1	AAACGGAGTCTCACACTGTC	
*MDM2*	MDM2ChIPF1	CATTTGGGTACAACTCCAGC	115
	MDM2ChIPR1	TGGAAACTGCGACAAATGCG	
*MYC*	MYCChIPF2	AAAGGGAGAGGGTTTGAGAG	226
	MYCChIPR2	GAGATTAGCGAGAGAGGATC	
*MCL1*	MCL1ChIPF2	CAACAGAGCTAGACTGTCTC	204
	MCL1ChIPR2	CACGTGCTACCCTAAAGAAC	
*STAT3*	STAT3ChIPF2	GCTGCTCTCCTCATTGGTC	258
	STAT3ChIPR2	CCTGTCCAGGATCCGGTTG	

### Statistical analysis

Results were presented as mean ± SD. Statistical analysis was carried out with Student's *t*-test, *P* < 0.05 was considered as statistically significant.

## SUPPLEMENTARY MATERIALS FIGURES AND TABLES


