# Supplementary material for: The epigenetic modifier CHD5 functions as a novel tumor suppressor for renal cell carcinoma and is predominantly inactivated by promoter CpG methylation
Source: Oncotarget. 2016 Mar 1;7(16):21618–30. doi: 10.18632/oncotarget.7822 (PMC5008310; doi:10.18632/oncotarget.7822)
Supplement: Supplementary file 1 [file oncotarget-07-21618-s001.pdf]

The epigenetic modifier CHD5 functions as a novel tumor suppressor for renal cell carcinoma and is predominantly inactivated by promoter CpG methylation

Supplementary Materials

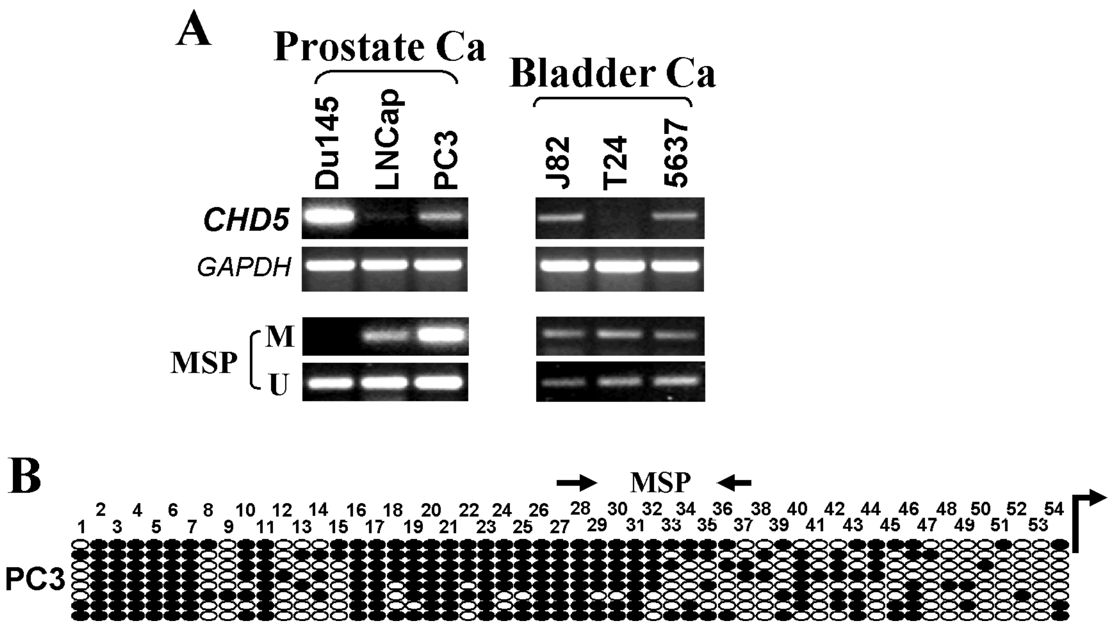

Supplementary Figure S1: (**A**) *CHD5* was downregulated or silenced by promoter methylation in prostate and bladder tumor cell lines as determined by RT-PCR and MSP. Ca, carcinoma. (**B**) BGS analysis of the *CHD5* promoter in PC3 cell line.

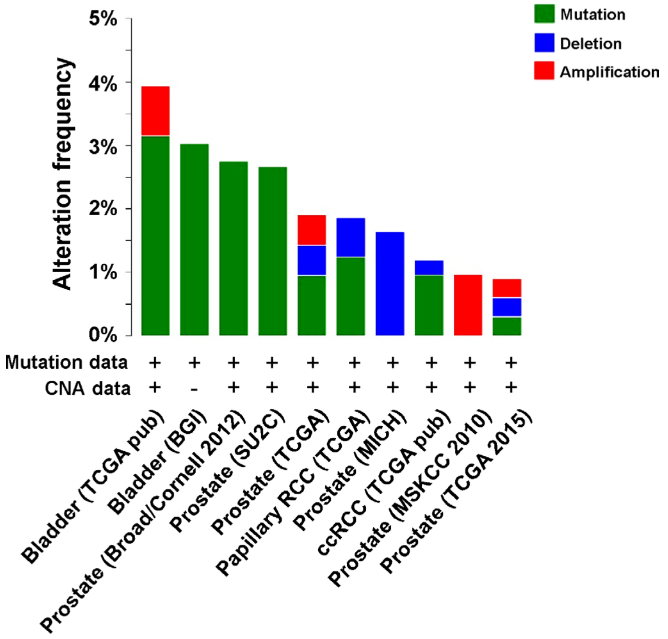

Supplementary Figure S2: *CHD5* is genetically altered in urological cancers, including mutation, deletion and amplification.

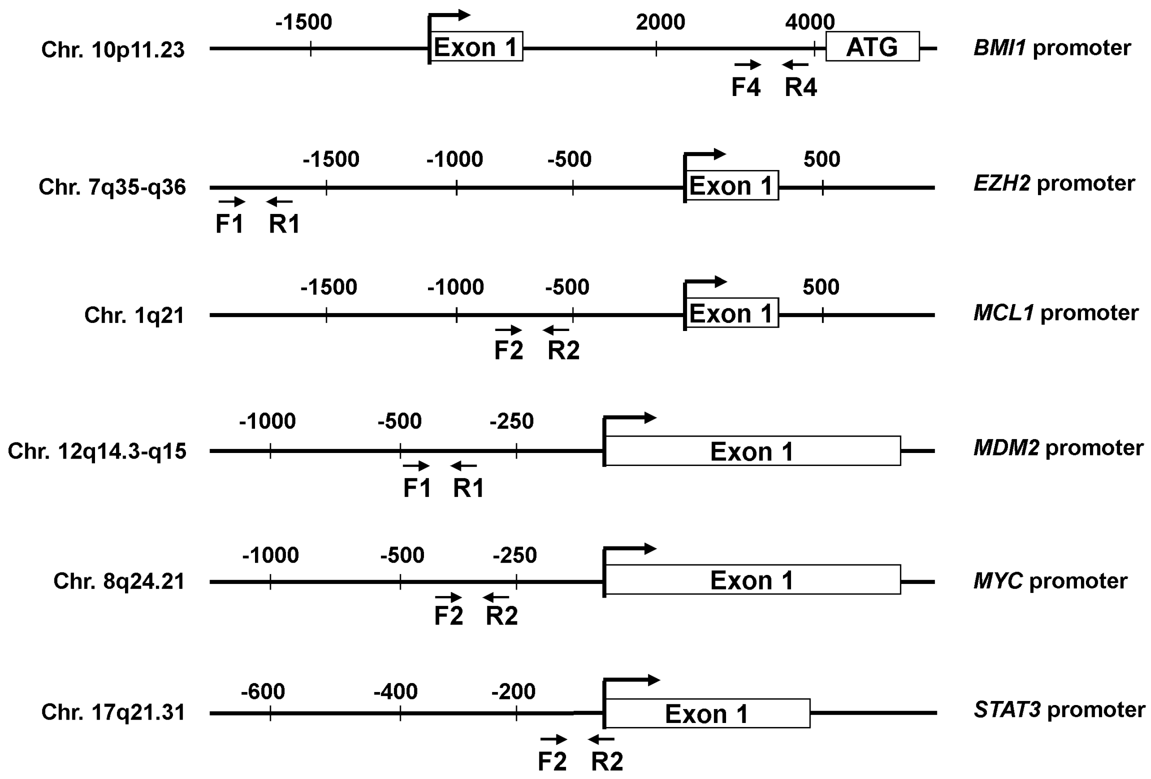

Supplementary Figure S3: Schematic of ChIP primers used in this study.

Supplementary Table S1: *CHD5* expression is significantly decreased in urological cancers

Cancer type		Median log2 mRNA (Range) in Normal Samples	Median log2 mRNA (Range) in Cancer Samples	*P*-Value	Ref.
Kidney	ccRCC	0 (−0.228 - 0.117) (*n* = 441)	−0.008 (−0.713 - 0.436) (*n* = 489)	3.06E–14	[31]
	Papillary RCC	0 (−0.228 - 0.117) (*n* = 441)	−0.128 (−0.631 - 0.157) (*n* = 27)	1.71E–5	
	Hereditary ccRCC	−1.537 (−1.713 - −1.375) (*n* = 10)	−1.738 (−1.967 - −1.423) (*n* = 32)	1.52E–4	[32]
	Non-Hereditary ccRCC	−1.537 (−1.713 - −1.375) (*n* = 10)	−1.704 (−1.971 - −1.283) (*n* = 27)	0.007	
	ccRCC	−1.095 (−1.215 - −0.915) (*n* = 9)	−1.179 (−1.299 - −1.11) (*n* = 9)	0.009	[33]
Bladder	Superficial Bladder Cancer	−0.586 (−1.395 - −0.223) (*n* = 9)	−1.542 (−2.152 - −0.751) (*n* = 28)	5.98E–6	[34]
	Infiltrating Bladder Urothelial Carcinoma	−0.586 (−1.395 - −0.223) (*n* = 9)	−1.393 (−1.865 - −1.043) (*n* = 13)	4.04E–5	
Prostate	Prostate Carcinoma	−0.354 (−0.776 - 0.025) (*n* = 9)	−0.787 (−1.365 - 0.494) (*n* = 25)	0.021	[35]

Supplementary Table S2: Somatic mutation spectrum of CHD5 in urological cancers

Sample ID	Cancer type	AA change	Type	COSMIC*	Copy	PolyPhen-2 Prediction	Ref.
B25	Bladder	K1180N	Missense	NA	NA	Probably Damaging	[41]
B112	Bladder	G1752V	Missense	NA	NA	Probably Damaging	[41]
B109	Bladder	H1716R	Missense	NA	NA	Benign	[41]
B109	Bladder	S714C	Missense	NA	NA	Probably Damaging	[41]
B112	Bladder	G1752C	Missense	NA	NA	Probably Damaging	[41]
TCGA-G2-A2EJ-01	Bladder	R584H	Missense	NA	Diploid	Probably Damaging	TCGA
TCGA-DK-A3IK-01	Bladder	V187I	Missense	NA	Diploid	Benign	[40]
TCGA-FD-A3SS-01	Bladder	R1618X	Nonsense	NA	Gain	Damaging	[40]
TCGA-DK-A1A3-01	Bladder	X1638_splice	Splice	NA	Diploid	Damaging	[40]
TCGA-A3-3382-01	ccRCC	L1768H	Missense	NA	Diploid	Probably Damaging	TCGA
TCGA-B0-4836-01	ccRCC	V45M	Missense	1	Diploid	Benign	[31]
TCGA-CJ-6031-01	ccRCC	N444K	Missense	NA	Diploid	Probably Damaging	[31]
TCGA-B0-4837-01	ccRCC	Y1733C	Missense	NA	Diploid	Probably Damaging	[31]
TCGA-EV-5902-01	pRCC	D1363E	Missense	NA	ShallowDel	Possibly Damaging	TCGA
TCGA-A4-7583-01	pRCC	K324N	Missense	NA	ShallowDel	Benign	TCGA
MO 1012	Prostate	G1908R	Missense	NA	Gain	Possibly Damaging	[39]
SC 9094	Prostate	A1822T	Missense	NA	Diploid	Possibly Damaging	[39]
TP 2061	Prostate	P941L	Missense	NA	Gain	Probably Damaging	[39]
6115114	Prostate	G1582R	Missense	NA	Diploid	Possibly Damaging	[39]
PR-04-639	Prostate	A1291V	Missense	NA	Diploid	Probably Damaging	[42]
P09-2497	Prostate	D1685Y	Missense	NA	Diploid	Possibly Damaging	[42]
PR-00-1165	Prostate	R1133H	Missense	NA	Diploid	Benign	[42]
TCGA-HC-8216-01	Prostate	F36I	Missense	NA	Gain	Benign	[38]
TCGA-KK-A59V-01	Prostate	R1621W	Missense	NA	Diploid	Probably Damaging	TCGA
TCGA-HC-7211-01	Prostate	R1906C	Missense	NA	Diploid	Probably Damaging	TCGA
TCGA-YL-A8S8-01	Prostate	R584H	Missense	NA	Diploid	Probably Damaging	TCGA

pRCC, Papillary RCC; *Mutation confirmed in COSMIC database; NA, not available.

Supplementary Table S3: Primers for screening target genes by semi-quantitative and quantitative RT-PCR

Gene	Primers	Sequence (5′–3′)	Length (bp)
BMI1	BMI1F	GATCACTGAGCTAAATCCCC	288
	BMI1R	CAGAAGGATGAGCTGCATAAA	
CCND1	CCND1F	TGCTGCGAAGTGGAAACCAT	243
	CCND1R	GCGGTCCAGGTAGTTCATG	
CHD1L	CHD1LF	ACTGCGGCTTCATACTGAGG	159
	CHD1LR	CCCAGGATACAGCCATTCTG	
DOT1L	DOT1LF	GGAGCTGAGACTGAAGTCGC	328
	DOT1LR	GACCTGCTGCAGGATATGGC	
EZH2	EZH2F	AGGGACCAGTTTGTTGGCG	262
	EZH2R	GGGATGACTTGTGTTGGAAAA	
EGFR	EGFRF	GTCCAGTATTGATCGGGAGA	303
	EGFRR	CACCTCCTGGATGGTCTTTA	
FN1	FN1F	AGGCTCAGCAAATGGTTCAG	251
	FN1R	AGGACGCTCATAAGTGTCAC	
HMGA1	HMGA1F	CCAGCGAAGTGCCAACACCT	166
	HMGA1R	GAGGACTCCTGCGAGATGCC	
HMGA2	HMGA2F	CACTTCAGCCCAGGGACAAC	207
	HMGA2R	CTAGGTCTGCCTCTTGGCCG	
HIF2A	HIF2AF	CGACAATGACAGCTGACAAG	188
	HIF2AR	GATTGCCAGTCGCATGATG	
HIG2	HIG2F	ACGACCTGCTCCTACAGC	177
	HIG2R	GAAGATGGAGAGTAGGGTCA	
ID1	ID1F	TGGAGATTCTCCAGCACGTC	181
	ID1R	ATGCGATCGTCCGCAGGAAC	
JMJD2C	JMJD2CF	AAGATAATGACCTTCAGACCC	255
	JMJD2CR	GAACTCCTTCACAGTCATCG	
MCL1	MCL1F	AGTTGTACCGGCAGTCGCTG	456
	MCL1R	CTAGGTCCTCTACATGGAAG	
MDM2	MDM2F	GCAATACCAACATGTCTGTAC	265
	MDM2R	CTTGGCACGCCAAACAAATC	
MYC	MYCF	CTCTCCGTCCTCGGATTCTC	211
	MYCR	GCCTCCAGCAGAAGGTGATC	
NANOG	NANOGF	ATGAGTGTGGATCCAGCTTG	190
	NANOGR	CCTGAATAAGCAGATCCATGG	
OCT4	OCT4F	AAGGAGAAGCTGGAGCAA	305
	OCT4R	GAGGGTTTCTGCTTTGCAT	
SNAI1	SNAI1F	CCAATCGGAAGCCTAACTAC	152
	SNAI1R	AGAGTCCCAGATGAGCATTG	
STAT3	STAT3F	CCAATGGAATCAGCTACAGC	236
	STAT3R	GCTGATAGAGAACATTCGACTC	
STAT5B	STAT5BF	ACAAGCTCAGCAGCTCCAAG	162
	STAT5BR	TGGGTGGCCTTAATGTTCTC	
VEGF	VEGFF	ACTTTCTGCTGTCTTGGGTG	311
	VEGFR	CTGCATGGTGATGTTGGACT	
YAP1	YAP1F	CATGAGGCTCCGGAAGCTGC	232
	YAP1R	CTGTCGAAGATGCTGAGCTG	
GAPDH	GAPDH33	GATGACCTTGCCCACAGCCT	302
	GAPDH55	ATCTCTGCCCCCTCTGCTGA	
